# Ultrasound Diagnosis of a Retained Migrated Foreign Body Following Penetrating Injury to The Upper Thigh in a Child: A Case Report Demonstrating an Underused Indication for Diagnostic Ultrasound

**DOI:** 10.24908/pocus.v7i2.15349

**Published:** 2022-11-21

**Authors:** Alexander Sheeka, Richard Jenkins, Sufi Sadigh, Nicholas Alexander, Afshin Alavi

**Affiliations:** 1 Department of Imaging, Imperial College Healthcare NHS Trust London United Kingdom; 2 Department of surgery and cancer, Imperial College London London United Kingdom; 3 Department of Paediatric Surgery, Imperial College Healthcare NHS Trust London United Kingdom

**Keywords:** Retained Foreign body, Penetrating Trauma, Delayed Diagnosis, Emergency Ultrasound

## Abstract

We present a case of delayed diagnosis of retained glass foreign body in the inguinal region of a child using ultrasonography following penetrating trauma to the upper thigh. The foreign body had traversed significantly by the time of diagnosis, from the medial upper thigh to the inguinal region at the level of the inguinal ligament. Ultrasound can be an effective initial imaging modality for the diagnosis of foreign bodies in children, allowing the potential to reduce ionizing radiation exposure.

## Case File

A 3 year-old boy of normal development and no previous medical history presented to his local emergency department with an incisional wound to the medial region of the right thigh following an unwitnessed injury involving the breaking of a glass vase. The wound was situated lateral to the right scrotum, V-shaped, with the underlying subcutaneous fat exposed. The child was treated with wound closure strips. No diagnostic imaging was performed at the time of presentation as there was a low clinical suspicion of retained foreign body given the wound appearance. 

**Figure 1:**
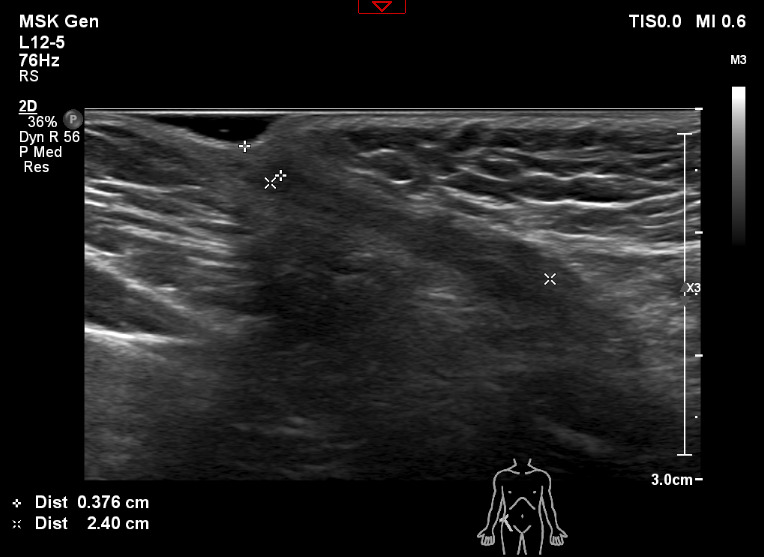
Longitudinal ultrasound image of the foreign body identified in the inguinal region.

**Figure 2:**
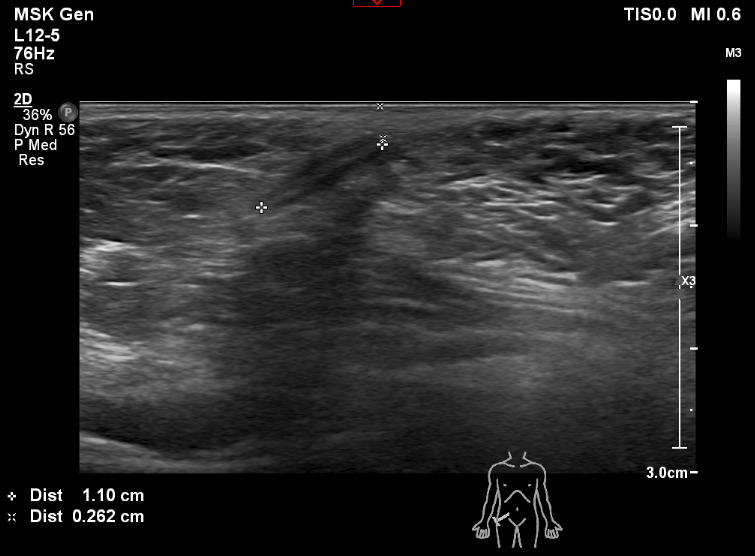
Transverse ultrasound image of the foreign body identified in the inguinal region.

The child re-attended the emergency department of a different hospital after 9 months due to continued right groin pain and progressive indentation of the inguinal skin. A diagnostic ultrasound scan was performed with a 12-5 MHz linear transducer by a diagnostic radiologist of greater than 3 years’ experience in pediatric ultrasound. The scan demonstrated a 2.4 x 1.1 cm foreign body 0.4 cm beneath the skin. The foreign body was hyperechoic with surrounding hypoechoic tissue and posterior acoustic shadow in keeping with a retained shard of glass (Figures 1 and 2).

**Figure 3:**
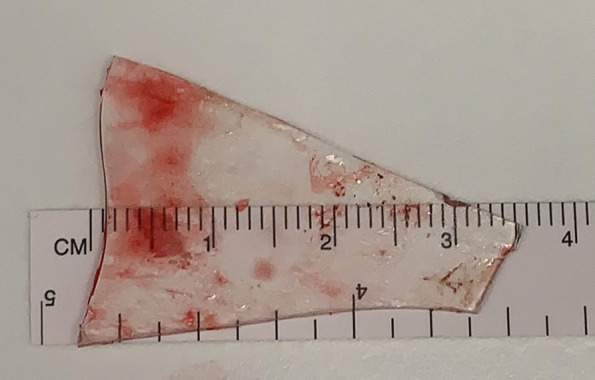
The 3.5 cm glass foreign body identified in the right inguinal region following surgical removal.

**Figure 4:**
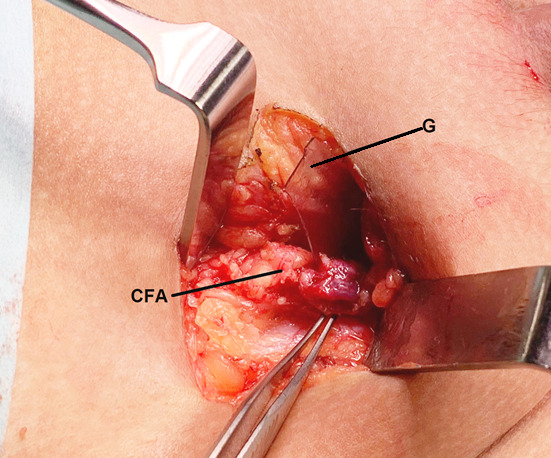
The glass foreign body (G) removed during open exploration of the right inguinal region. The shard was identified abutting the common femoral artery (CFA) and had transected the vas deferens. (Cranial end to the left of the image).

The patient was taken to theater for surgical exploration and removal of the foreign body. A 3.5 x 2.3 cm glass shard (Figure 3) was found abutting the right common femoral vessels roughly 6 cm proximal to the original thigh wound (Figure 4). Exploration of the inguinal region demonstrated a transected vas deferens which was unable to be repaired at the time of surgery. The child was clinically improved post-operatively with reduced pain and improved mobility. 

 

## Discussion

In the pediatric population penetrating injuries are uncommon and their study is largely limited to observational and retrospective studies 1. As a result, much of the assessment criteria for the pediatric population is extrapolated from adult studies 2. Assessment in children following trauma is further complicated in that while adult populations have a high correlation between foreign body sensation and foreign body presence 3, the correlation is not present in case series involving children 4. This increases the importance of imaging to aid diagnosis.

Penetrating foreign bodies are well documented in adults; in some cases, the objects have migrated significant distances from the site of trauma both at the time and after the original injury 5. Although not commonly documented in children, foreign body migration is particularly concerning given the changing anatomy of the growing child. These changes may increase the risk of migration and predispose to serious adverse clinical outcomes, such as our presented case, where the glass foreign body was found abutting the femoral artery and had transected the vas deferens.

Plain radiography is the most common method to assess for soft-tissue foreign bodies, and multiple studies have confirmed it as a sensitive method of assessment for radiopaque objects, reaching 100% sensitivity for metal, and 96% for glass 6. However, plain radiograph has the disadvantages of utilizing ionizing radiation, being insensitive to radiolucent objects such as wood, and being inaccurate in determining involvement of the surrounding soft tissue structures, particularly the neurovasculature. 

The use of ultrasound in assessing the presence of soft tissue foreign bodies has been the subject of several studies with varying conclusions. A meta-analysis on the role of ultrasound in foreign body detection demonstrated a pooled sensitivity and specificity of 72% and 92% respectively 7. Studies comparing the subjective assessment of image quality in ultrasound, plain radiograph, and tomographic imaging amongst radiologist cohorts supported the inter-observer reliability of ultrasound to identify foreign bodies in muscle and bone 8. Ultrasound was particularly advantageous in cases where the foreign body is thought to be radiolucent, outperforming plain radiographs. Contrary to these findings, other studies have shown that the pooled sensitivity and specificity are significantly lower, and ultrasound should only be considered an adjunct to imaging modalities such as plain radiograph 9, 10. The sonographic appearances of different foreign bodies are well characterized, including many of the compositions of commercial glass 11. Both laminated glass (used in automotives) and soda-lime glass (used in domestic bottles and containers) demonstrate high echogenicity with posterior shadow as was seen in the presented case.

One particular concern for the use of ultrasound in the assessment of foreign bodies is the associated inflammation and hemorrhage caused by acute trauma, its effect on image quality, and subsequently diagnostic accuracy. Reviews of the sonographic characteristics of soft tissue foreign bodies in the acute phase suggest that hemorrhage, edema, and granulation tissue cause a hypoechoic rim around the foreign body which distinguishes it from the surrounding soft tissue 12. A hypoechoic rim around the foreign body in our case likely represented granulation tissue, and was visible even outside the acute phase.

To assess the temporal development of soft tissue changes due to the presence of a foreign body, a study utilizing an anesthetized porcine model found that only 7% of foreign bodies demonstrated surrounding soft tissue edema within two hours of insertion. The sensitivity and specificity of identification of wood, metal, and glass with ultrasound immediately post-insertion was 85% and 86% respectively. This increased to 87% and 89% respectively after 2 hours, indicating high diagnostic accuracy in the acute phase even without the presence of surrounding soft tissue changes 13. 

There are several limits to the use of ultrasound in foreign body detection. One major limitation affecting ultrasound procedures is inter-operator variability. Although some studies suggest variability is low^8^ the case presented was diagnosed by an experienced pediatric radiologist. Accuracy in detecting foreign bodies may vary depending on operator experience and the quality of the machine used. Furthermore, foreign bodies adjacent to gas-filled or calcified structures are more difficult to identify due to adjacent artefact. 

## Conclusion

There is a recognized risk of foreign body retention in children following penetrating trauma, with potential for the foreign body to traverse significant distances from the original site of trauma, making a prompt diagnosis difficult on clinical examination alone. Ultrasound is underused in the diagnosis of foreign bodies, however it can be potentially used as an initial diagnostic imaging modality or as an adjunct in surgical planning, providing detailed soft tissue assessment not provided by plain radiography. 

## Statement of Consent

The Parents of the child in the report have given verbal and written consent to the publication of the associated images.

## Disclosures

None.

## References

[R165879626892911] Cotton B, Nonce M (2004). Penetrating trauma in children. Semin Pediatr Surg.

[R165879626892908] Haines E, Fairbrother H (2019). Evaluation and Management of Pediatric Patients with Penetrating Trauma to the Torso. Pediatr Emerg Med Pract.

[R165879626892904] Tahmasebi M, Zareizadeh H, Motamedfar A (2014). Accuracy of Ultrasonography in Detecting Radiolucent Soft-Tissue Foreign Bodies. Indian J Radiol Imaging.

[R165879626892901] Friedman D I, Forti R J, Wall S P, Crain E F (2005). The Utility of Bedside Ultrasound and Patient Perception in Detecting Soft Tissue Foreign Bodies in Children. Pediatr Emerg Care.

[R165879626892900] Ozsarac M, Demircan A, Sener S (2011). Glass Foreign Body in Soft Tissue: Possibility of High Morbidity due to Delayed Migration. J Emerg Med.

[R165879626892905] Anderson M A, Newmeyer W L, Kilgore E S (1982). Diagnosis and Treatment of Retained Foreign Bodies in the Hand. Am J Surg.

[R165879626892899] Davis J, Czerniski B, Au A, Adhikari S, Farrell I, Fields J M (2015). Diagnostic Accuracy of Ultrasonography in Retained Soft Tissue Foreign Bodies: A Systematic Review and Meta-Analysis. Acad Emerg Med.

[R165879626892909] Aras M H, Miloglu O, Barutcugil C, Kantarci M, Harorli A (2010). Comparison of the Sensitivity for Detecting Foreign Bodies Among Conventional Plain Radiography, Computed Tomography and Ultrasonography. Dentomaxillofac Radiol.

[R165879626892906] Ranieri D De, Lin S (2021). Applications of Musculoskeletal Ultrasound in the Pediatric Emergency Department. Pediatr Ann.

[R165879626892910] Chianca V, Pietto F Di, Zappia M, Albano D, Messina C, Sconfienza L M (2020). Musculoskeletal ultrasound in the emergency department. Semin Musculoskelet Radiol.

[R165879626892907] Horton L K, Jacobson J A, Powell A, Fessell D P, Hayes C W (2001). Sonography and Radiography of Soft-Tissue foreign bodies. AJR Am J Roentgenol.

[R165879626892902] Jacobson J A, Powell A, Craig J G, Bouffard J A, Holsbeeck M T Van (1998). Wooden Foreign Bodies in Soft Tissue: Detection at US. Radiology.

[R165879626892903] Turandot S, Siadecki S D, Rose G, Berkowitz R, Matilsky D, Godbold J, Moshier E (2015). Ultrasound Accurately Identifies Soft Tissue Foreign Bodies in a Live Anesthetized Porcine Model. Acad Emerg Med.

